# Supplementary material for: Are carbon emissions trading and green financial instruments synergistic? -Comprehensive quantitative research based on content analysis
Source: PLoS One. 2024 Mar 7;19(3):e0298601. doi: 10.1371/journal.pone.0298601 (PMC10919601; doi:10.1371/journal.pone.0298601)
Supplement: S1 Data — (ZIP) [file pone.0298601.s001.zip › Data/地级市Z_F工作报告.zip!/░▓╗╒/╕╖╤⌠╩╨/└┤╫╘╣1⁄2╓┌║┼í╛┬φ┐╦╩2╛▌═°í┐_2018.docx]

各位代表：

　　现在，我代表市人民政府，向大会报告政府工作，请予审议，并请市政协委员和其他列席人员提出意见。

　　一、2017年工作回顾

　　过去的一年，在省委、省政府和市委的坚强领导下，全市上下坚持以习近平新时代中国特色社会主义思想为指导，全面学习贯彻党的十八大和十九大精神，认真贯彻落实省委、省政府和市委决策部署，大力实施“双轮驱动”战略，扎实推进“五大专项行动”，主动作为，攻坚克难，开拓奋进，全市经济社会继续保持了平稳较快发展的良好态势，顺利完成了市五届人大一次会议确定的目标任务。

　　——经济增长势头强劲。1-11月份，全市固定资产投资等6项经济指标增幅居全省首位。在省政府第三季度经济运行考核中，位居第一。预计全年生产总值1600亿元，比上年增长9%。财政收入277.1亿元，增长22.6%、增幅居全省首位，总量前移一位、迈入第一方阵。

　　——转型升级步伐加快。1-11月份，全市战略性新兴产业产值、高新技术产业增加值分别增长32.6%和30.8%，均居全省首位。太和现代医药产业集聚发展基地产值增长25%。江淮中重卡投产运营，煤基新材料产业园一期项目即将投产。全社会研发投入增长21.3%，新增高新技术企业29家、省级院士工作站7家，分别达到148家和9家。

　　——城乡建设加速推进。阜城建设完成投资386亿元，创历史最高水平，增长40.4%。拆违拆旧专项行动取得突破性进展，全市完成征迁3200万平方米、增长55%，其中阜城1500万平方米，是上年的1.6倍。

　　——脱贫攻坚成效显著。“十大工程”年度任务全面完成，“四带一自”产业扶贫模式在全省推广。全省产业扶贫、“千企帮千村”精准扶贫和易地扶贫搬迁现场会在我市召开。19.2万人脱贫、170个贫困村出列的任务可望顺利实现。

　　——民计民生持续改善。33项民生工程任务全面完成，全年投入资金159.2亿元，城乡居民人均可支配收入持续增长。基层医改“阜南模式”成为全省样板，国家义务教育基本均衡发展实现县域全覆盖。

　　一年来，我们重点抓了以下几个方面的工作：

　　（一）突出工业主导，大力发展实体经济。坚持把发展工业作为实体经济的核心，组织实施工业转型升级专项行动，工业经济提质增效。1-11月份，规模以上工业增加值增长10.2%、居全省第二位，单位工业增加值能耗下降13.4%。新增规模以上工业企业142家、省级“专精特新”企业35家。国家级高新区、经开区创建工作全面启动。现代生态农业产业化步伐加快。粮食总产567.8万吨，连续10年超百亿斤。土地流转552万亩，规模养殖比重达79%。新增农民专业合作社1535家、家庭农场3200家。专用品牌小麦基地发展到93万亩，农产品加工业产值突破1000亿元。颍上县入围首批国家农业可持续发展试验区。现代服务业蓬勃发展。实现社会消费品零售总额850亿元，增长12%。阜阳农产品物流中心全面建成。电子商务进农村在全省率先实现全覆盖，电商主体发展到9500家，交易额达800亿元。旅游业总收入164亿元，增长20.9%。阜阳机场航线增加到14条，旅客吞吐量突破60万人次，增长35.6%。金融机构贷款余额2040.5亿元，增长33.1%。

　　（二）突出阜城带动，加快推进城乡建设。坚持把中心城市建设摆在更加突出的位置，全力推进阜城大投入、大建设、大发展。高度重视规划引领，“多规合一”规划编制完成，专项规划进一步完善。城南新区建设快速推进，西湖新区、高铁新区启动建设，阜城建成区扩大到130平方公里，城市框架进一步拉大。实施道路桥梁提升行动，滨河路、一道河路西延和东环路、向阳路颍河桥建成通车，解放北路、航颍路泉河桥加快建设，城市功能进一步完善。五县市规划建设管理全面加强，75个乡镇政府驻地建成区整治和71个中心村建设进展顺利。城乡环境整治专项行动扎实推进，阜城14条黑臭水体治理初见成效，主干道路建筑立面改造完成680万平方米。扎实开展农村垃圾处理、污水治理、厕所改造“三大革命”，改造农村厕所9.5万户，乡镇街道、村居环卫保洁基本实现市场化运营。绿化提升专项行动成效明显，植树造林投入24.5亿元、创历史新高，完成成片造林12万亩，阜城新增绿地4.1万亩，城市形象进一步提升。

　　（三）突出扩大投资，持续增强发展后劲。坚持把扩大有效投入作为加快发展的主动力，大力实施全民招商专项行动，建立项目推进“四项机制”，组织开展重点项目推进、重点项目编制两个“百日会战”，累计谋划入库项目1388个，总投资额达1万亿元。项目投资规模明显扩大，亿元以上重点项目完成投资1106亿元，预计全年固定资产投资1630亿元、增长26%。商阜杭、郑阜高铁和铁路北站扩能工程加快建设，淮河阜阳港、引江济淮工程和阜阳机场改扩建工程前期工作进展顺利。开工建设国省干线公路277公里，完成农村道路畅通工程1991公里，农村电网改造完成投资12.8亿元。水利基础设施建设完成投资36亿元，创历史新高。精准开展系列招商活动，实际到位省外亿元以上非房地产项目资金514亿元，增长24.7%。华大基因、新松机器人、深圳光启等行业领军企业项目成功落户。

　　（四）突出深化改革，加速释放发展活力。坚持把改革作为破解发展难题的关键之举，全面深化供给侧结构性改革，切实推进减税降费，全年结构性减税56亿元，降低企业费用13亿元。持续深化“放管服”改革，大力推广“互联网+政务服务”，编制政务服务事项实施清单1205项，建成市、县一体化网上政务服务平台。新一轮户籍制度改革深入实施，农业转移人口落户城镇4.4万人。深入推进商事制度改革，全年新增市场主体7.9万户、增长21.4%，均居全省第二位。全面完成城市综合执法体制改革，整合相关职能近200项。 农村“三变”改革和土地承包经营权抵押贷款试点步伐加快，农村集体土地颁证全面完成。金融改革稳步推进，“新三板”和省股权交易中心挂牌企业新增114家，居全省首位。国资国企和教育、卫生、文化等领域改革也都取得了新的进展。

　　（五）突出精准扶贫，全面打好年度战役。坚持把脱贫攻坚作为重大政治任务和第一民生工程，围绕“保一争一”目标，扎实开展“回头看、找差距、补短板、促提升”等专项行动，以“绣花”功夫推进脱贫攻坚“十大工程”。制定落实加强行蓄洪区脱贫攻坚政策措施，建成5个省级就业扶贫基地、119个市级就业扶贫基地，新建改建扶贫车间639个，完成易地扶贫搬迁3937人、贫困户危房改造4.2万户。健全完善“三保障一兜底一补充”一站式医疗结算报销体系，“351”“180”健康扶贫政策有效落实，贫困户实际医疗支出大幅降低。建成农村饮水安全工程147处，解决了237.3万人饮水安全问题。

　　（六）突出民生为本，全力维护和谐稳定。坚持以人民为中心的发展思想，大力推进基本公共服务均等化。切实加强大气、水、土壤等污染综合防治，扎实开展中央、省环保督察突出问题整改，环境质量进一步改善。高度重视就业创业，深入开展“接您回家”系列活动，全年新增城镇就业6.9万人，3.1万农民工返乡创业，创办实体2.9万个。深化机关事业单位养老保险、社会保险制度改革，城乡低保月均补差分别增长14.7%和31.8%。阜阳一中、阜阳十中新校区和阜阳七中加快建设，新建公办幼儿园24所。市三院、五院、六院新区和安医大附属阜阳医院建成开诊。市市民中心、档案馆、城市规划展示馆建成使用，市博物馆、科技馆、图书馆和大剧院加快建设。精神文明建设成效显著，我市荣获“全国文明城市提名城市”、颍上县荣获“全国文明城市县级提名城市”荣誉称号。6人当选或提名全国、全省道德模范，17人被评为“中国好人”“安徽好人”，位居全省前列。成功举办市第十三届运动会、第三届安徽省剪纸艺术节暨第二届阜阳文博会、第四届安徽省民间杂技艺术节。注重加强和创新社会治理，深入开展“守护平安”系列行动，大力实施化解信访积案专项工程，严格落实安全生产责任制，切实加强食品药品安全监管，人民群众安全感和满意度进一步提升。国防动员和人防建设工作深入推进。妇女、儿童及老龄、老干部工作进一步加强。民族、宗教、统计、档案、地方志、气象、地震、外事、侨务、台湾事务等工作也都取得了新的成绩。

　　一年来，我们落实全面从严治党责任，扎实推进“两学一做”学习教育和“讲、重、作”专题教育，严格执行向市委报告工作制度，政府系统党的建设得到加强。深入推进依法行政，规范政府决策行为，自觉接受人大法律监督、工作监督和政协民主监督，全年办理人大代表建议190件、政协委员提案323件。出台了政府规章《阜阳市户外广告和招牌设置管理办法》《阜阳市房屋建筑和市政基础设施工程质量管理办法》。大力推进政府行政效能建设，健全抓落实的工作机制，认真办理市长热线、市长信箱和网友留言。加强政务督查和目标管理绩效考核，持续深化“严、转、提、促”活动。认真抓好省委第二巡视组反馈意见整改落实。强化行政监察和审计监督。切实加强党风廉政建设，严格执行中央八项规定精神和省、市相关要求，营造了风清气正、干事创业的良好氛围。

　　各位代表！过去的一年，成绩来之不易，必须倍加珍惜。这是省委、省政府和市委正确领导的结果，是市人大、市政协大力支持的结果，是全市上下凝心聚力、锐意进取、扎实苦干的结果。在此，我代表市人民政府，向在各个岗位上辛勤工作的全市人民，向给予政府工作大力支持的人大代表、政协委员，向各民主党派、工商联、各人民团体和社会各界人士，向驻阜解放军指战员、武警官兵和政法干警，向所有关心支持阜阳发展的同志们、朋友们，表示衷心的感谢！

　　在肯定成绩的同时，我们也清醒地看到，我市经济社会发展中仍存在一些困难和问题。一是发展不足的问题依然突出，经济总量不大、质量不高，人均水平偏低，实体经济水平亟待提升。二是基本公共服务水平较低，贫困人口相对较多，稳定脱贫和防范返贫的压力较大。三是生态环保和维护稳定任务艰巨，大气污染防治形势严峻，社会治理有待加强。四是发展环境有待优化，少数干部推动新发展的能力不足，专业素养还有待提高，不作为、慢作为的现象还不同程度地存在。对此，我们一定高度重视，下大力气认真解决，绝不辜负人民的期待。

　　二、2018年工作安排

　　今年是贯彻落实党的十九大精神的开局之年，是改革开放40周年，是决胜全面建成小康社会、实施“十三五”规划承上启下的关键一年，做好全年各项工作意义重大。当前阜阳正处于决战脱贫攻坚、决胜全面小康的关键期，加速追赶、转型跨越的黄金发展期，乘势而上、大有可为的战略机遇期。我们既有着人口消费红利、建设投资市场巨大、交通区位优越等比较优势，又有着国家实施乡村振兴和脱贫攻坚战略、省加快皖北地区振兴等政策机遇。同时，全市上下政治生态风清气正，经济发展势头强劲，社会大局和谐稳定，干部队伍精神饱满。只要我们锐意进取，扎实苦干，就一定能够开创阜阳加快发展的崭新局面！

　　今年政府工作的总体要求是：全面贯彻党的十九大精神，以习近平新时代中国特色社会主义思想为指导，以新发展理念统领发展全局，坚持稳中求进工作总基调，围绕高质量发展根本要求，以供给侧结构性改革为主线，以五大发展行动计划为总抓手，认真落实市委五届六次全会暨经济工作会议决策部署，深入实施“双轮驱动”战略，扎实推进“五大专项行动”，统筹做好稳增长、促改革、调结构、惠民生、防风险各项工作，推动质量变革、效率变革、动力变革，着力在打好防范化解重大风险、精准脱贫、污染防治的攻坚战方面取得扎实进展，切实提高保障和改善民生水平，促进经济社会又好又快发展。

　　经济社会发展的主要预期目标是：全市生产总值增长9%，财政收入增长10%，固定资产投资增长16%，规模以上工业增加值增长10%，社会消费品零售总额增长11%，城镇、农村常住居民人均可支配收入分别增长8.5%和9%，城镇调查失业率控制在5%以内，完成省下达的节能减排任务。

　　实现上述目标，突出抓好九项重点工作。

　　（一）坚定不移实施工业突破，大力提升新型工业化水平

　　深入推进工业转型升级专项行动，实施工业发展三年行动计划，统筹推进传统产业改造升级和新兴产业引进培育，着力壮大工业经济规模。

　　加大传统产业改造提升力度。依托规模以上工业企业，加大技术改造投入，引导绿色食品、煤电化工、再生资源综合利用等传统产业转型升级，滚动实施一批新技术应用、新产品开发、新模式嵌入和“机器人换人”项目，努力打造千亿元产业、百亿元企业。加快煤基新材料、中重卡汽车制造、秸秆综合利用环保等产业园建设，开工建设金种子科技酿酒二期工程、阜阳卷烟厂易地技改、华润电厂二期工程等重点项目，力争全年新增规模以上工业企业100家，省级“专精特新”企业30家。

　　加大新兴产业引进培育力度。大力推进“三重一创”建设，打造一批具有重要影响力的战略性新兴产业集群。积极发展智能制造产业，促进互联网、大数据、人工智能与制造业深度融合，加快推进新松机器人和北斗大数据产业园建设。积极发展生命健康产业，加强与华大基因战略合作，建设国家基因库阜阳资源库，加快太和现代医药产业集聚发展基地建设，完善上下游产业链，做大规模、打响品牌。着力提升节能环保产业，加大绿色建筑产业培育力度，进一步提升城市矿产综合利用和集聚发展水平。

　　加大开发园区转型升级力度。突出产业聚集，培育园区首位产业，努力形成主业突出、特色鲜明、错位发展的园区建设新格局。突出功能配套，进一步完善基础设施，开展闲置低效用地清理，提升园区综合承载能力。突出品牌创建，对各类开发园区进行清理、整合、提升，落实“一县一区”。着力促进阜阳经济技术开发区、颍州经济开发区、阜合现代产业园区整合创建国家级经开区，以界首市高新区为主体创建国家级阜阳高新区，支持阜南县和太和县创建国家级经开区，支持临泉县建设省级电子信息产业基地。

　　加大科技创新推进力度。制定出台创新型城市创建方案，推广界首科技创新经验，启动全市创建工作。支持企业创建升级技术研发中心和建立院士工作站、博士后流动站、研究生实践基地，组建一批技术创新战略联盟，打造以企业为主体、市场为导向、产学研深度融合的产业技术创新平台。围绕价值链中高端产业，构建研究院、产业园、基金群、政策包为一体的支撑体系，全社会研发投入增长20%以上。积极实施普惠制科技创新券制度，充分调动科技型中小企业和创新团队、创客的创新积极性。高度重视智慧经济发展，依托光启阜阳智慧城市研究院，一体推进研发攻关、产品应用和产业培育。在城市管理、医疗、教育、养老等领域开展“智慧+”应用试点示范，积极打造智慧产业新高地。实施质量品牌战略，推进质量强市建设。

　　（二）坚持不懈推进城市建设，全面加速新型城镇化进程

　　立足打造区域性中心城市，实施城市建设三年行动计划，深入推进拆违拆旧、城乡环境整治专项行动，加快建设现代化大美阜阳。

　　持续放大中心城市能级。切实把阜城建设摆上更加突出的位置，全面加快城市建设步伐，力争全年完成投资400亿元以上。进一步发挥规划引领作用，推进“多规合一”实施，持续优化城市空间布局，推进城市快速交通等各类专项规划编制。进一步拓展城市规模，重点推进城南新区、西湖新区、高铁新区和颍东、颍泉拓展区建设。进一步完善城市路网，加快建设航颍路泉河桥、解放北路泉河桥和颍柳路颍河桥，拆除重建阜裕大桥，推进向阳北路、淮河路、新韩路、西湖大道、环湖大道等路网工程。进一步完善城市功能，加快推进市博物馆、科技馆、图书馆和大剧院建设，配套完善社区综合体、养老医疗、文体活动场所等公共服务设施，打造内容丰富、方便快捷的生活圈。启动新一轮三年棚改攻坚计划，新开工棚户区改造安置房53711套，基本建成30827套、公租房2136套。

　　不断提升城市形象。以创建全国文明城市为重点，同步推动全国绿化模范城市、国家园林城市、省卫生城市、省绿色生态城市创建工作，出台年度工作方案，大力组织实施，全方位提升城市品质。加快建设颍河景观带、七渔河防震减灾公园、植物园、金种子生态文化产业园等一批公园游园。深入推进以“三项整治”为主要内容的市容环境综合治理，持续开展主干道路楼体立面整治，全面推行清扫保洁、垃圾处理和园林养护市场化。大力发展公共交通，加强交通秩序整治，着力缓解交通拥堵现象。进一步加大公共停车场建设力度，逐步解决停车难、停车乱等突出问题，塑造文明有序、卫生整洁的良好形象。

　　切实加强县城建设。按照“四创一建”要求，深入推进五县市规划建设管理，加快建设道路、管网等基础设施，不断完善学校、医院等公共服务设施，加强特色塑造、环境改造，努力打造宜居宜业、特色鲜明的中等城市。按照彰显特色、打响名片的要求，强化县城产业支撑，选准县域经济发展路径，有效提升县域经济规模和综合竞争力。

　　打造现代基础设施体系。实施综合交通建设和综合治水三年行动计划。加快商阜杭、郑阜高铁建设，开工建设阜阳至霍邱至合肥高速公路，加快阜阳至淮滨、沿淮高速前期工作。新建国省干线410公里、农村道路2100公里。加速推进阜阳复线船闸、颍上复线船闸工程。扎实做好机场改扩建前期工作，持续开辟新的航线。加快推进铁路北站扩能、阜阳铁公水综合物流港、淮河阜阳港项目。全面完成新一轮中小河流治理和大中型涵闸除险加固，加快引江济淮、引淮济阜和洪汝河、泉河治理等工程建设，不断完善城乡一体现代基础设施体系。

　　（三）大力实施乡村振兴战略，着力推进农业农村现代化

　　坚持农业农村优先发展，按照产业兴旺、生态宜居、乡风文明、治理有效、生活富裕的总要求，科学制定乡村振兴战略规划，建立健全城乡融合发展体制机制和政策体系，加快推进农业农村现代化。

　　扎实推进农村综合改革。尽快建成县级土地承包经营权信息应用平台，推进农村土地所有权、承包权、经营权“三权”分置。积极推动农村集体产权制度改革，扩大“三变”改革试点范围。加强农民教育和农村人才培养，让种地农民成为职业农民、务工农民成为技能人才、返乡农民成为创业主体，充分调动“三元”主体积极性。强化社会化服务，提升适度规模经营水平，促进小农户和现代农业发展有机衔接，新建现代农业产业化联合体70个。深化新一轮户籍制度改革，有效促进农业转移人口市民化和农民就地城镇化。

　　扎实推进现代生态农业产业化建设。巩固提升粮食产能，专用品牌粮食扩大到200万亩。重点发展阜南奶羊、颍上奶牛、临泉肉羊等规模生态健康养殖，突出发展市场需求的蔬菜、花卉等高效经济作物，增加绿色品牌农产品供给。规划建设沿淮河、沙颍河、泉河、黑茨河、苏沟和阜南至颍上6大花卉产业带，力争全市花卉面积达到50万亩。以特色小镇建设为引领，培育现代休闲农业特色优势产业带和产业群。支持现有中小型农产品加工企业做大做强，培育以福中和为代表的一二三产业融合发展品牌化龙头企业，打造农产品加工业“甲级队”，力争农产品加工业产值增长15%以上。

　　扎实推进美丽宜居乡村建设。全面完成75个乡镇政府驻地和71个中心村建设任务，启动新一轮141个中心村建设。持续开展农村“三大革命”，完善垃圾收集、转运、处理体系，加强乡镇政府驻地和美丽乡村中心村生活污水治理设施建设，完成自然村改厕26万户。切实加强“四好”农村公路建设，实施一批加宽改造、路面硬化项目。巩固提升农村饮水安全工程，提高农村自来水普及率、集中供水率和水质达标率。开展新一轮农村电网改造升级，推进城乡电网融合发展，提升农村供电质量。坚持自治、法治、德治相结合，弘扬乡村美德，推进乡风文明，让农民安居乐业、农村长治久安。

　　（四）加快发展现代服务业，着力促进消费转型升级

　　积极培育大市场、发展大物流、拓展新业态，全面提升现代服务业发展档次和水平，巩固提升区域性商贸物流中心地位。

　　积极培育消费新增长点。推进传统商圈升级改造，健全社区商业综合服务体系，加快各类专业市场建设。巩固电子商务进农村全覆盖成果，力争规模以上、限额以上企业电商应用率达80%。积极适应消费升级，重点发展文化、体育、健康、养老等幸福产业，推动技术服务、供应链物流、研发设计、人力资源培训等生产性现代服务业蓬勃兴起。加快发展会展经济，广泛开展经贸会展活动，精心打造一批具有影响力的本地展会品牌。

　　强力推进旅游业大发展。科学编制全域旅游发展规划，提升阜阳旅游整体竞争力。实施核心景区提升工程，推进八里河景区和祥源颍淮生态文化旅游区提升等重点项目，启动颍州西湖景区起步区开发建设，挖掘历史文化，完善功能配套，打造具有影响力的休闲度假旅游目的地。实施乡村旅游提升工程，打造乡村旅游精品线路，发展民宿客栈、采摘篱园、特色农庄，培育一批乡村旅游节庆品牌。加强旅游商品开发，拉动旅游购物消费，力争旅游业年收入突破200亿元。

　　努力实现外贸企稳回升。充分发挥出口退税“周转池”和中小进出口企业专项担保基金作用，推动外贸优进优出。完善海铁联运软硬件配套，做好铁路一类口岸、水运二类口岸和进口肉类指定口岸申报工作，申建公用型保税仓库、出口监管仓库，积极加入“合新欧”班列，促进外贸自由化便利化。

　　（五）健全完善项目服务机制，持续扩大有效投资规模

　　坚持经济工作项目化、项目工作责任化，不断健全完善项目推进“四项机制”， 始终把对外开放作为第一战略、招商引资作为第一抓手，深入推进全民招商专项行动，切实增强投资对经济增长的支撑力。

　　强化项目谋划编制。按照保主保重保急的原则，紧盯国家宏观政策导向和资本要素流向，重点围绕五大发展行动计划、“双轮驱动”战略和“五大专项行动”，在产业升级、基础设施、生态环保、民生改善等领域，精心谋划一批重大项目。进一步做实做细项目前期工作，加大申报推介力度，形成滚动增加、梯次推进的良性循环。

　　强化项目投资融资。突出招商引资主渠道作用，出台新一轮招商引资政策，坚持精准算账、精准识别、精准对接、精准服务，全面提升招商引资质量和水平，确保全年引进市外资金增长15%以上、直接利用外资增长10%以上。充分发挥城投、交投、工投、科投等政府投融资平台作用，提高运营质量和效益。充分利用企业上市“绿色通道”机遇，推进企业上市。加大银企对接力度，保持贷款增长的良好势头。积极发展混合所有制经济，激发民间资本活力，多措并举扩大投资规模。

　　强化项目建设管理。坚持政府引导、市场运作、建管结合、一体推进，开展全域土地整治和集约节约利用行动计划，做好城乡建设用地增减挂钩，为项目落地争取更多土地空间。优化土地征迁机制，开展精准拆迁，完善招标投标机制和运营管理机制，做到精准对接、规范透明、高效廉洁，为项目建设提供优良环境。

　　强化项目调度推进。围绕市级重大项目，认真落实市、县领导联系重点项目制度和“四督四保”推进机制。发挥基建类、产业类两个市级项目调度指挥中心作用，推行项目信息化台账管理和动态管控。完善重点项目建设目标考核办法，实行每月一调度、每季一现场、半年一观摩、每年一奖惩，切实推进项目建设提速提质提效。

　　（六）不断深化重点领域改革，全面释放经济发展活力

　　突出重点领域和关键环节，增强改革定力，狠抓改革落地，破解发展难题，最大限度激发市场活力和社会创造力。

　　深化供给侧结构性改革。认真落实“三去一降一补”任务，制定实施处置“僵尸企业”方案，加快淘汰落后产能，坚决破除无效供给，为新产业发展腾出空间。严格实行住宅房价备案和预售审批，着力将房价稳定在理性范围内，加大非住宅商品房去库存力度，促进房地产市场平稳健康发展。持续推进“四送一服”双千工程，全面落实领导联系包保机制，切实打通降成本优惠政策落实“最后一公里”。

　　深化“放管服”改革。统筹考虑各类机构设置，深化机构和行政体制改革，推进事业单位分类改革，筹建数据资源局、花卉产业发展局，开展市公共资源交易监管机构改革试点。进一步简政放权，创新监管方式，推进商事制度改革。围绕打造“四最”营商环境，持续减少审批事项，完善“互联网+政务服务”体系，实现政府数据资源共享，推进“一网通办”和“不见面”审批，提升市民中心服务功能，营造稳定、公平、透明、可预期的营商环境，让政务服务更智慧、群众办事更便捷、创新创业更顺畅。

　　深化人才机制改革。实行更加积极、更加开放、更加有效的人才政策，探索建立市政府特殊津贴制度，积极推行柔性管理政策，健全编制“周转池”管理制度，鼓励高层次人才创新创业和自由流动。促进高等、中等职业教育资源整合，培育知识型、技能型、创新型劳动者大军，统筹推进技工大市、科教大市和制造强市建设。充分激发和保护优秀企业家精神，着力构建“亲”“清”新型政商关系，在全社会营造尊重企业家、服务企业家、宽容企业家的良好环境，让各类市场主体在颍淮大地创业愉快、发展愉快、生活愉快。

　　深化财政金融改革。继续深化预算编制改革，不断完善全口径预算管理体系。推进市、区财政事权和支出责任划分改革，全面实施预算绩效管理。规范政府举债融资机制，加强政府债务管理，有效防控债务风险。建立健全金融风险监测预警、早期干预和应急处置机制，加大非法集资案件处置力度，严厉打击互联网金融领域违法行为，有效遏制金融风险。积极引进股份制商业银行和保险、证券、基金等非银行机构。加快发展普惠金融，持续推进城乡金融服务均等化。同时，按照一企一策、分类推进的原则,加快国企改革步伐。推进经营性国有资产集中统一监管，确保国有资产效益最大化和效率最优化。

　　（七）切实加大精准扶贫力度，坚决打赢脱贫年度战役

　　坚持把脱贫攻坚作为重大政治任务和第一民生工程，确保实现19.3万人脱贫、158个村出列，颍上县、太和县、界首市、颍州区、颍泉区5个县市区摘帽。

　　加快补齐基本公共服务短板。重点围绕医疗、教育、住房等基本公共服务8个主要领域，针对薄弱环节、重点人群，不断提升基层基本公共服务能力和水平。严格执行“351”“180”健康扶贫和社保兜底扶贫政策。加强贫困地区学前教育，实现建档立卡贫困家庭学生资助全覆盖。扎实推进农村危房改造和易地扶贫搬迁工程，统筹解决搬迁后可持续发展问题。

　　全面落实产业扶贫关键举措。重点围绕产业发展，大力实施贫困村“一村一品”产业推进行动，全面推广“四带一自”产业扶贫模式，引导新型经营主体与贫困村、贫困户建立紧密的利益联结机制，实现产业扶贫全覆盖。新建扶贫车间531个、就业扶贫驿站120个，建设51个乡村旅游扶贫重点村。大力发展资产收益扶贫，推进土地承包经营权等入股参与企业生产，积极开发保险扶贫“特惠保”产品，增加贫困群众财产性收入。

　　切实加大深度贫困地区攻坚力度。重点围绕阜南、颍上、临泉三县沿淮行蓄洪区、洪洼地区等重点贫困地区，整合各方要素资源，认真落实新增脱贫攻坚资金、项目、举措向深度贫困地区倾斜的政策，大力支持发展适应性特色产业，把低洼地的劣势转化为产业发展的优势。加快推进村（庄）台、保庄圩整治，谋划实施一批居民迁建、环境治理、基础设施、生活保障等重点项目，改善群众生产生活条件。

　　健全完善工作落实责任体系。重点围绕今年脱贫攻坚作风建设年的要求，深入开展扶贫领域作风问题专项治理，坚决杜绝弄虚作假、搞数字脱贫。严格落实党政一把手负总责的责任制，完善市、县、乡、村四级责任清单，实现责任链闭环管理、无缝对接。坚持扶贫与扶志、扶智相结合，健全防范返贫预警机制，稳定脱贫后续帮扶措施。落实村专干工资待遇，打造一支懂扶贫、会帮扶、作风硬的扶贫干部队伍。积极开展扶贫日活动和扶贫志愿者行动，持续推进“百企帮百村”，凝聚社会扶贫强大合力。

　　（八）着力加强生态文明建设，推动环境质量持续改善

　　坚持生态立市、绿色崛起，牢固树立和践行绿水青山就是金山银山的理念，严守生态保护、永久基本农田和城市开发边界红线，努力建设天蓝、水清、地绿的大美阜阳。

　　大力实施颍淮蓝天工程。认真落实中央大气污染防治新的行动计划，实行大气污染防治网格化精准监测，做到线上预警和线下监管相结合。不断强化扬尘、烟尘、粉尘治理，落实烟花爆竹禁燃措施。加快秸秆综合利用收储运体系建设，培育引进一批龙头企业，进一步提升秸秆综合利用水平。切实改善大气环境质量，努力减少重度污染天数，不断增强人民群众的蓝天幸福感。

　　扎实推进水和土壤污染防治。推动淮河等重点流域水污染综合治理，高度重视并着力解决湖泊污染问题。加快城市黑臭水体治理，组织实施农村黑臭水体排查治理，加大污水管网和雨污分流设施建设力度，提升城乡生活污水治理设施处理能力和运营效率，切实保障饮用水源安全。开展土壤污染状况详查和污染地块修复试点，推进畜禽养殖废弃物综合利用，构建草地河湖休养生息长效机制。

　　深入推进绿化提升专项行动。按照增加绿量、打造精品、建管并重的原则，加快实施一批城乡绿化项目，打造一批园林绿化精品工程，不断提升绿化档次和水平，力争阜城人均公共绿地面积达到9平方米。实施沙颍河生态景观带、颍州西湖湿地保护区建设，打造生态修复特色功能区。大力发展生态林业和民生林业，完成成片造林5万亩，创建森林长廊示范段70公里、省级森林城镇4个，持续推进林业增绿增效。

　　积极发展绿色循环低碳经济。树立绿色设计、建造、运营理念，推广节能环保新技术、新工艺、新装备。积极发展新能源和可再生能源，促进资源综合利用和循环利用，深入推进国家级循环经济示范城市创建工作。强化全民生态文明意识，开展创建节约型机关、绿色家庭、绿色学校、绿色社区和绿色出行等活动，让尊重自然、呵护生态成为全市人民的自觉行动。

　　建立完善生态环境管理制度。认真落实能源、水资源、建设用地总量和强度“双控”制度。严格执行排污许可制度，探索开展碳排放权交易试点。全面落实河长制、林长制。持续推进中央、省环保督察突出问题整改。严格执行领导干部任期生态文明建设责任制、自然资源资产离任审计制度和生态环境损害责任终身追究制。探索实施环境信用联合激励和惩戒措施，坚决制止和惩处破坏生态环境行为。

　　（九）全面发展社会各项事业，有效保障改善民计民生

　　坚持尽力而为、量力而行，大力推进基本公共服务均等化，精准实施33项民生工程，着力提高民生工程质量效益，让改革发展成果更多更公平惠及全市人民。

　　优先发展教育事业。进一步深化教育体制综合改革，大力推动城乡义务教育优质均衡发展，切实解决中小学生课外负担重、“择校热”“大班额”等问题。顺应新一轮高考招生制度改革，加强教学设施和师资队伍建设。加快建设阜阳一中新校区，建成阜阳十中新校区和阜阳七中。大力发展学前教育，实施第三期学前教育行动计划，着力解决婴幼儿照护和儿童早期教育服务问题。实施第二期特殊教育提升计划。规范引导民办教育发展。大力发展职业教育，启动职业教育三年提升计划，加快实施阜阳职业技术学院新区项目。支持创建阜阳师范大学，促进阜阳师范学院信息工程学院独立发展，启动阜阳技师学院二期、阜阳幼儿师范专科学校二期建设。

　　加强就业和社会保障工作。完善就业创业服务体系，深化拓展“接您回家”系列活动，落实优惠扶持政策，吸引更多大学生、研究生、科研人员、企业家等群体支持家乡建设。大力实施城乡居民持续增收工程。积极开发公益性岗位，加强就业援助。健全城乡居民和城镇职工基本养老保险制度，完善新型城乡社会救助体系、医疗救助制度和临时救助制度。

　　推进健康阜阳建设。深化医药卫生体制综合改革，全面推广“阜南模式”，大力推进医共体、医联体建设，完善分级诊疗机制，提高家庭医生签约服务覆盖率。提升计划生育服务管理水平，促进人口长期均衡发展。推进产前无创基因检测唐氏综合征、耳聋和遗传代谢病检测全覆盖。探索医养结合新模式，推动居家养老、社区养老、机构养老衔接发展，加快构建多层次养老服务体系。

　　推动文化事业繁荣发展。持续实施公民道德建设工程，广泛宣传社会主义核心价值观，加大时代楷模、道德模范、先进典型、最美人物宣传力度，进一步打响“阜阳好人”品牌。

积极开展精神文明创建活动，倡导健康文明生活方式。深化“书香阜阳”全民阅读活动。扎实推进文化惠民工程，丰富群众性文化体育活动。切实强化宣传引领，加快推进传统媒体与新兴媒体融合发展，着力提高新闻舆论传播力、引导力、影响力、公信力。加强文化遗产传承保护利用。持续深化文化体制改革，推动文艺精品创作和文化品牌建设。

　　提高社会治理水平。认真落实重大决策社会稳定风险评估机制，加大矛盾纠纷排查化解力度。进一步畅通信访渠道，切实解决群众合理诉求，及时化解信访积案。深入开展“铸安”行动，加强食品药品安全全过程监管，有效防范和坚决遏制重特大事故的发生。加强应急平台和专业队伍建设，提高应急保障能力。不断深化平安阜阳建设，完善社会治安防控体系，依法严厉打击各类违法犯罪行为，强化互联网安全监管，进一步提升人民群众安全感和满意度。推动智慧社区建设，推行网格化管理，加强和创新基层社会治理。促进社会诚信体系建设，营造优良信用环境。深入开展法制宣传教育，推进“七五”普法。健全农村留守儿童和老年人关爱帮扶体系。加强统计工作基层基础，做好第四次全国经济普查工作。促进少数民族和民族聚居地区加快发展，着力提高依法管理宗教事务水平。加强国防动员、后备力量建设和人民防空工作，推动军民深度融合发展，积极争创全国双拥模范城。高度重视国家安全、保密工作。支持工会、共青团、妇联、科协和关工委、老龄委、老干部工作。做好档案、气象、地震、地方志和外事、侨务、台湾事务等工作。发展红十字、残疾人和福利慈善事业。

　　三、加强政府自身建设

　　新时代要有新气象，更要有新作为。各级政府及其工作人员要顺应时代发展要求，不断提高政府工作现代化水平，努力为全市人民创造更加美好的生活。

　　（一）强化政治意识，不断提升科学施政能力。全面学习贯彻党的十九大精神，以习近平新时代中国特色社会主义思想武装头脑、指导实践、推动工作。进一步增强“四个意识”，严守政治纪律、政治规矩，自觉在思想上政治上行动上同以习近平同志为核心的党中央保持高度一致，坚定不移地落实好省委、省政府和市委各项决策部署。切实担负起新时代的历史使命，必须增强“八种本领”。推动经济高质量发展，还要着力提升创新引领能力、招商引资能力、服务企业能力和运用金融能力。

　　（二）强化法治意识，不断提升依法行政能力。切实把依法行政作为政府工作的基本准则，严格依照法定权限和程序履行职责。加强政府立法制度建设，完善合法性审查提前介入机制，基本实现村（居）委会法律顾问全覆盖。主动接受人大法律监督、工作监督和政协民主监督，认真办理人大代表建议和政协委员提案。广泛听取各民主党派、工商联、无党派人士和各人民团体的意见，主动接受司法监督、群众监督、舆论监督，加强政府内部层级监督和专门监督，让权力在阳光下运行。

　　（三）强化宗旨意识，不断提升执政为民能力。坚持把执政为民的要求融入到思想感情里，体现到政策措施中，落实到具体行动上，确保各项工作更加符合群众意愿。把老百姓的安危冷暖时刻放在心上，以造福人民为最大政绩，着力解决群众普遍关注的教育、就业、收入、社保、医疗、养老、居住、环境等方面的事情。健全完善领导干部联系和服务群众机制，大力推进网络问政和网上办事，切实办好市长热线、市长信箱和网友留言，积极回应群众关切，依法保障人民群众权益。

　　（四）强化效能意识，不断提升高效服务能力。积极践行“一线工作法”，深入开展调查研究和现场办公。持续深化“严、转、提、促”活动，大力弘扬“五干”作风，完善保障工作落实的闭环链条，实行重大项目全程监测、实时调度，提高一次部署完成率。牢固树立强烈的担当意识，在困难面前迎难而上、提高斗志、奋发有为，在矛盾面前挺身而出、敢抓敢管、绝不回避，在改革面前大胆探索、勇于创新、砥砺前行。进一步完善政府目标管理绩效考核体系，建立健全激励机制和容错纠错机制，推动形成想作为、敢作为、善作为的良好风尚。

　　（五）强化廉洁意识，不断提升拒腐防变能力。始终把纪律和规矩挺在前面，切实履行党风廉政建设主体责任，支持纪检监察机关依法依规履行职责，坚定不移严明法纪、严惩腐败，推动干部清正、政府清廉、政治清明。深化廉政风险防控，加强对财政资金使用、公共资源交易、国有资产管理等重点领域的监督管理，推进审计监督全覆盖，把权力关进制度的笼子。严格执行中央八项规定及实施细则精神和省、市相关要求，一以贯之改进工作作风，驰而不息整治“四风”问题。全体政府工作人员尤其是领导干部要时刻保持蓬勃朝气、昂扬锐气、浩然正气，以实际行动赢得广大群众的信任、拥护和支持。

　　各位代表！幸福都是奋斗出来的。让我们在中共阜阳市委的坚强领导下，不忘初心、牢记使命，撸起袖子加油干，甩开膀子使劲干，确保完成今年经济社会发展目标任务，为加快建设现代化大美阜阳而努力奋斗！
